# RapC Is Involved in the Development of *Dictyostelium* Through the Regulation of Autophagy and Calcium Signaling

**DOI:** 10.3390/cells15201878

**Published:** 2026-10-15

**Authors:** Nara Han, Uri Han, Dahyeon Kim, Wonbum Kim, Taeck Joong Jeon

**Affiliations:** 1 Department of Integrative Biological Sciences & BK21 FOUR Educational Research Group for Age-Associated Disorder Control Technology, Chosun University, Gwangju 61452, Republic of Korea; 2 The Basic Science Institute of Chosun University, Chosun University, Gwangju 61452, Republic of Korea

**Keywords:** RapC, Rap GTPase, TORC1, *Dictyostelium*, autophagy

## Abstract

RapC is a Rap-subfamily small GTPase in *Dictyostelium* whose developmental function remains largely uncharacterized. Upon starvation, individual amoebae aggregate and undergo morphogenetic transitions to ultimately produce a fruiting body, a process requiring precise spatiotemporal regulation of a single apical tip as the primary organizing center. Here, we report that RapC is required for tip organizer formation during multicellular development by modulating autophagy and intracellular calcium signaling. *rapC* null cells develop normally through aggregation but consistently form three to four apical tips per aggregate rather than the single organizer tip characteristic of wild-type cells. Pharmacological phenocopy experiments revealed that caffeine and the selective TORC1 inhibitor rapamycin recapitulate the multi-tip phenotype, whereas the PI3K inhibitor LY294002 and the pan-TOR inhibitor Torin2 do not, implicating TORC1 signaling as the relevant downstream effector. In line with this, *rapC* null cells exhibit normal GFP-PH:CRAC translocation kinetics and slightly elevated PKB/PKBR1 phosphorylation, ruling out defects in the PI3K–TORC2–PKB signaling axis. Instead, *rapC* null cells display markedly elevated autophagic flux as assessed by the tandem RFP-GFP-Atg8 reporter assay, consistent with attenuated TORC1-mediated autophagy suppression. Furthermore, aggregation-competent *rapC* null cells show increased intracellular calcium ([Ca^2+^]i) upon external calcium stimulation, a response absent in wild-type cells and not reproduced by rapamycin, indicating a TORC1-independent role for RapC in calcium homeostasis. Together, our findings establish that RapC promotes normal *Dictyostelium* development by supporting TORC1 activity to suppress excessive autophagic flux and by maintaining [Ca^2+^]i homeostasis through a TORC1-independent mechanism.

## 1. Introduction

*Dictyostelium discoideum* is a eukaryotic model organism uniquely suited to the study of cAMP-driven chemotaxis, multicellular morphogenesis, and intracellular signal transduction [1]. Upon nutrient depletion, solitary amoebae secrete periodic cAMP pulses that act as both a chemoattractant and developmental morphogen through the GPCR cAR1, triggering Ras GTPase activation, PI3K-mediated production of phosphatidylinositol (3,4,5)-trisphosphate (PIP3), and activity of adenylyl cyclase A (ACA), the *Dictyostelium* enzyme responsible for cAMP synthesis. Pleckstrin homology (PH) domain-containing proteins are recruited to the plasma membrane by binding PIP3. The PH domain of cytosolic regulator of adenylyl cyclase (CRAC), fused to GFP (GFP-PH:CRAC), is therefore widely used as a live-cell reporter of local PIP3 production. The resulting cAMP relay drives aggregation of up to 100,000 cells, which undergo morphogenetic transitions through slug formation and culmination to produce a fruiting body of stalk cells and dormant spores [1,2].

A defining feature of slug morphogenesis is the formation of a single apical tip that functions as the dominant organizing center, coordinating multicellular patterning through oscillatory cAMP relay waves while suppressing secondary organizing centers via lateral inhibitory signals [1,2,3]. Disruption of tip dominance produces a characteristic multi-tip phenotype, documented across diverse genetic and pharmacological contexts—including loss of DdRasGAP1, deletion of 5′-nucleotidase (5nt), truncated TagA protease activity, SunB disruption, and caffeine treatment [4,5,6,7,8]. Despite this mechanistic diversity, how nutrient sensing and metabolic state are integrated with tip dominance remains incompletely understood.

The TOR (Target of Rapamycin) kinase operates within two functionally distinct complexes in *Dictyostelium* [9]. TORC2 phosphorylates PKBA and PKBR1 to relay chemotactic signals and drive morphogenesis, while TORC1 acts as the master negative regulator of autophagy by suppressing the Atg1/ULK1 initiation complex [10,11,12,13]. Notably, the TORC1/AMPK balance has been proposed to govern prestalk versus prespore cell fate decisions, with low TORC1 activity promoting an AMPK-high state associated with prestalk identity [10,13].

The Rap GTPase subfamily in *Dictyostelium* includes Rap1 (RapA), RapB, and RapC. Rap1 is well-characterized as a regulator of integrin-like cell–substrate adhesion, myosin II-dependent cortical tension, and TORC2-PKB chemotaxis signaling [12,14]. RapC shares 51% amino acid identity with RapA in its Ras domain but bears a unique extended C-terminal sequence [15]. Whereas RapA promotes cell–substrate adhesion and reduces chemotaxis speed, RapC acts antagonistically to suppress adhesion and facilitate rapid migration [16]. *rapC* null cells exhibit enhanced adhesion, reduced chemotaxis speed with intact directional sensing, cytokinesis defects, and—critically—multi-tip formation during slug development [15,16]. However, the downstream signaling mechanisms by which RapC governs multicellular morphogenesis have remained entirely undefined.

Autophagy delivers cytoplasmic components to the lysosome for degradation and recycling, and is required for normal *Dictyostelium* development; its dysregulation impairs slug and fruiting body formation [17,18]. Autophagic flux can be quantitatively resolved using the tandem RFP-GFP-Atg8 reporter, wherein autophagosome–lysosome fusion quenches GFP fluorescence in the acidic lumen, leaving red-only (RFP^+^/GFP^−^) puncta as a readout of flux [19]. Calcium (Ca^2+^) likewise plays broad regulatory roles in *Dictyostelium*, coupling with cAMP oscillations to coordinate multicellular patterning [20]. In yeast, starvation-triggered vacuolar Ca^2+^ release activates autophagy via a calmodulin–Rck2–Atg11 cascade [21], suggesting a conserved Ca^2+^–autophagy axis whose existence in *Dictyostelium* development has not been examined.

In this study, we address the molecular mechanisms by which RapC controls *Dictyostelium* multicellular morphogenesis. Through systematic pharmacological dissection of signaling pathways, PI3K/PKB activity profiling, autophagic flux analysis, and live-cell calcium imaging, we demonstrate that RapC governs development through two mechanistically distinct effector pathways: a TORC1-dependent pathway that suppresses autophagy to maintain single-tip organizer dominance, and a TORC1-independent pathway that maintains intracellular [Ca^2+^]i homeostasis in aggregation-competent cells. These findings establish a novel mechanistic framework connecting Rap GTPase signaling to nutrient-sensing and calcium regulatory networks during multicellular morphogenesis.

## 2. Materials and Methods

### 2.1. Cell Strains

*Dictyostelium discoideum* wild-type KAx3 cells were grown axenically in HL5 medium with shaking at 150 rpm or in association with *Klebsiella aerogenes* at 22 °C. Knockout strains and transformants were maintained in 10 µg/mL blasticidin or 10 µg/mL G418. *rapC* null cells and GFP-RapC rescue cells were described previously [15]. Disruption of the *rapC* gene in the null strain used here was confirmed previously by RT-PCR, and the specificity of the resulting phenotypes was validated by full rescue upon re-expression of GFP-RapC [15,16]. *acaA* null (DBS0235562), which lacks adenylyl cyclase A (ACA), and *pi3k1/2* null strains (DBS0236766) were obtained from the Dicty Stock Center (dictybase.org). A tandemly tagged autophagic marker, pRFP-GFP-Atg8 (G90681) [19], and a calcium reporter, pHK12(neo)-Dd GCaMP6s (G90729) [22], were obtained from the NBRP Cellular Slime Molds (NBRP Nenkin, Tsukuba, Japan).

### 2.2. Development

Development analysis was performed as described previously [15]. Exponentially growing cells were washed twice in 12 mM Na/K phosphate buffer (pH 6.1) and resuspended at a density of 3.5 × 10^7^ cells/mL. 50 µL of the cells were plated and developed in non-nutrient agar plates. For pharmacological experiments, inhibitors were incorporated into agar at the following concentrations: caffeine 2 mM (Sigma-Aldrich, St. Louis, MO, USA), LY294002 30 µM (Sigma-Aldrich), Torin2 1 µM (ApexBio, Houston, TX, USA), rapamycin 50 nM (MCE, Monmouth Junction, NJ, USA). Developmental morphology was photographed and examined with a stereomicroscope at the indicated time points in the figures. The number of tips per aggregate was counted at 14 h in more than 50 aggregates per condition across at least three independent experiments. Data are presented as mean ± SD.

### 2.3. Quantification of Cortical GFP-PH:CRAC and Intracellular Calcium Dynamics

Quantification of cortical GFP-PH:CRAC was performed as described previously [23]. Aggregation-competent cells were prepared by pulsing with 50 nM cAMP at 6 min intervals for 5–6 h in DB buffer at a density of 5 × 10^6^ cells/mL with constant shaking (150 rpm). Aggregation-competent cells expressing GFP-PH:CRAC were plated on glass-bottom dishes, allowed to adhere for 15 min, and uniformly stimulated with 15 µM cAMP. Time-lapse fluorescence images were acquired at 2 s intervals for 1 min using a fluorescence microscope equipped with NIS-Elements 3.0 software (Nikon, Tokyo, Japan). Cortical fluorescence intensity was analyzed using ImageJ 1.54p. software (National Institutes of Health, Bethesda, MD, USA). The cortical fluorescence intensity was determined by dividing the intensity at each time point (Et) by the intensity before stimulation (Eo). Data represent mean ± SD from at least three independent experiments.

Intracellular calcium dynamics using GCaMP6 were analyzed as described previously [24]. Vegetative or aggregation-competent cells expressing GCaMP6 were plated on glass-bottom dishes and uniformly stimulated with 10 mM CaCl_2_ (external calcium stimulus) or 15 µM cAMP (chemoattractant stimulus). For rapamycin experiments, cells were pre-incubated with 50 nM rapamycin for 30 min before stimulation. GCaMP6 fluorescence was recorded at 2 s intervals for 1 min and quantified as Et/E0 using the ROI manager in ImageJ. Data represent mean ± SD from at least three independent experiments.

### 2.4. Autophagic Flux Assay Using RFP-GFP-Atg8

Wild-type and *rapC* null cells stably expressing a tandemly tagged autophagic marker RFP-GFP-Atg8 [19] were imaged during exponential growth in HL5 or after 4 h of starvation in DB buffer. Fluorescence images were acquired using a fluorescence microscope. Scale bar = 100 µm. Autophagic cells were defined as cells containing red-only puncta, indicative of autolysosome formation. The percentage of autophagic cells and the number of puncta per cell were quantified in three independent experiments, each scoring more than 100 cells per condition. Data are presented as mean ± SD.

### 2.5. Immunoblotting and Statistical Analysis

Aggregation-competent cells were stimulated with 15 µM cAMP for 0 or 60 s and immediately lysed in SDS sample buffer. Proteins were resolved by 10% SDS-PAGE, transferred to PVDF membranes, and probed with the following primary antibodies: anti-phospho-Akt substrate (#9614; Cell Signaling, Danvers, MA, USA; 1:5000) to detect PKB substrate phosphorylation; anti-phospho-p70 S6 Kinase (#9206; Cell Signaling; 1:10,000) to detect PKBR1 hydrophobic motif phosphorylation at T470; and anti-β-actin (sc-47778; Santa Cruz, Dallas, TX, USA; 1:10,000) as loading controls. For inhibitor studies, cells were pre-treated with 1 µM Torin2 or 50 nM rapamycin for 30 min prior to stimulation. Band intensities were quantified by densitometry using ImageJ.

All data were collected from at least three independent experiments and expressed as mean ± standard deviation (SD). Statistical comparisons between two groups were performed using an unpaired two-tailed Student’s *t*-test. Multiple group comparisons were assessed by one-way ANOVA with Tukey’s post hoc test. A *p*-value of less than 0.05 was considered statistically significant. NS, not significant. For each figure, the number of independent experiments and, where applicable, the number of aggregates or cells scored are indicated in the corresponding figure legend.

## 3. Results

### 3.1. rapC Null Cells Exhibit a Multi-Tip Phenotype During Development

**Figure 1:**
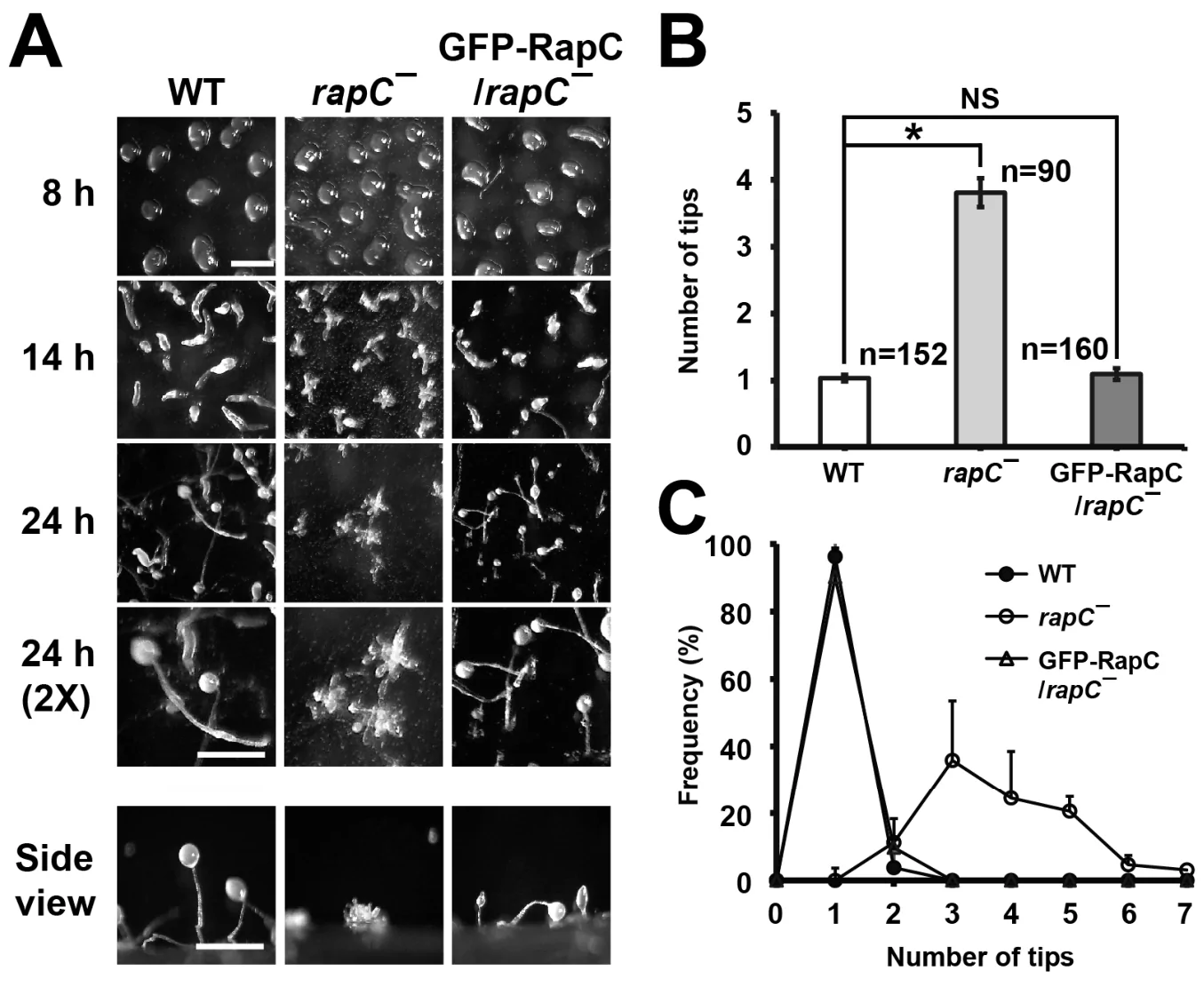
*rapC* null cells exhibit a multi-tip phenotype during development. (**A**) Development of wild-type (WT), *rapC* null (*rapC*^−^), and GFP-RapC-expressing *rapC* null cells (GFP-RapC/*rapC*^−^). Exponentially growing cells were developed on non-nutrient agar plates. Representative images at 8, 14, and 24 h are shown. A magnified (2×) view of a portion of the 24 h image is shown directly below the corresponding panel. Side view of fruiting bodies at 24 h development. Scale bar = 0.5 mm. (**B**) Average number of tips per aggregate at 14 h. The graphs show the mean number of tips counted from multiple aggregates across three independent experiments. The number of aggregates analyzed (N) is indicated on each graph. Error bars indicate SD. One-way ANOVA with Tukey’s post hoc test was performed. NS, not significant; * *p* < 0.05, statistically significant. (**C**) Frequency distribution of the number of tips per aggregate at 14 h. The percentage of aggregates bearing each tip number is shown.

To characterize the developmental role of RapC, we examined the developmental morphology of wild-type (KAx3), *rapC* null, and GFP-RapC-rescued *rapC* null cells on non-nutrient agar. Wild-type cells followed the expected developmental progression: loose aggregates at 8 h, single-tipped mounds at 12–14 h, and mature fruiting bodies at 24 h (Figure 1A). *rapC* null cells developed normally through aggregation but consistently formed multiple discrete tips per aggregate at 14 h (Figure 1A) [15]. Quantification at 14 h revealed an average of 3–4 tips per aggregate in *rapC* null cells compared to a single tip in wild-type cells (Figure 1B). Distribution analysis confirmed that while more than 90% of wild-type aggregates maintained a single tip, *rapC* null aggregates displayed a broad rightward distribution with a large proportion bearing three or more tips (Figure 1C).

By 24 h, *rapC* null cells produced multi-stemmed developmental structures arising from a single aggregate, rather than the single fruiting body of wild-type cells. Stable expression of GFP-RapC in *rapC* null cells completely rescued the single-tip morphology and normalized the tip-number distribution (Figure 1A–C), confirming that the phenotype is caused specifically by loss of RapC. These results establish RapC as a novel and essential regulator of tip dominance during *Dictyostelium* mound morphogenesis.

### 3.2. Caffeine Treatment Phenocopies the rapC Null Multi-Tip Phenotype

**Figure 2:**
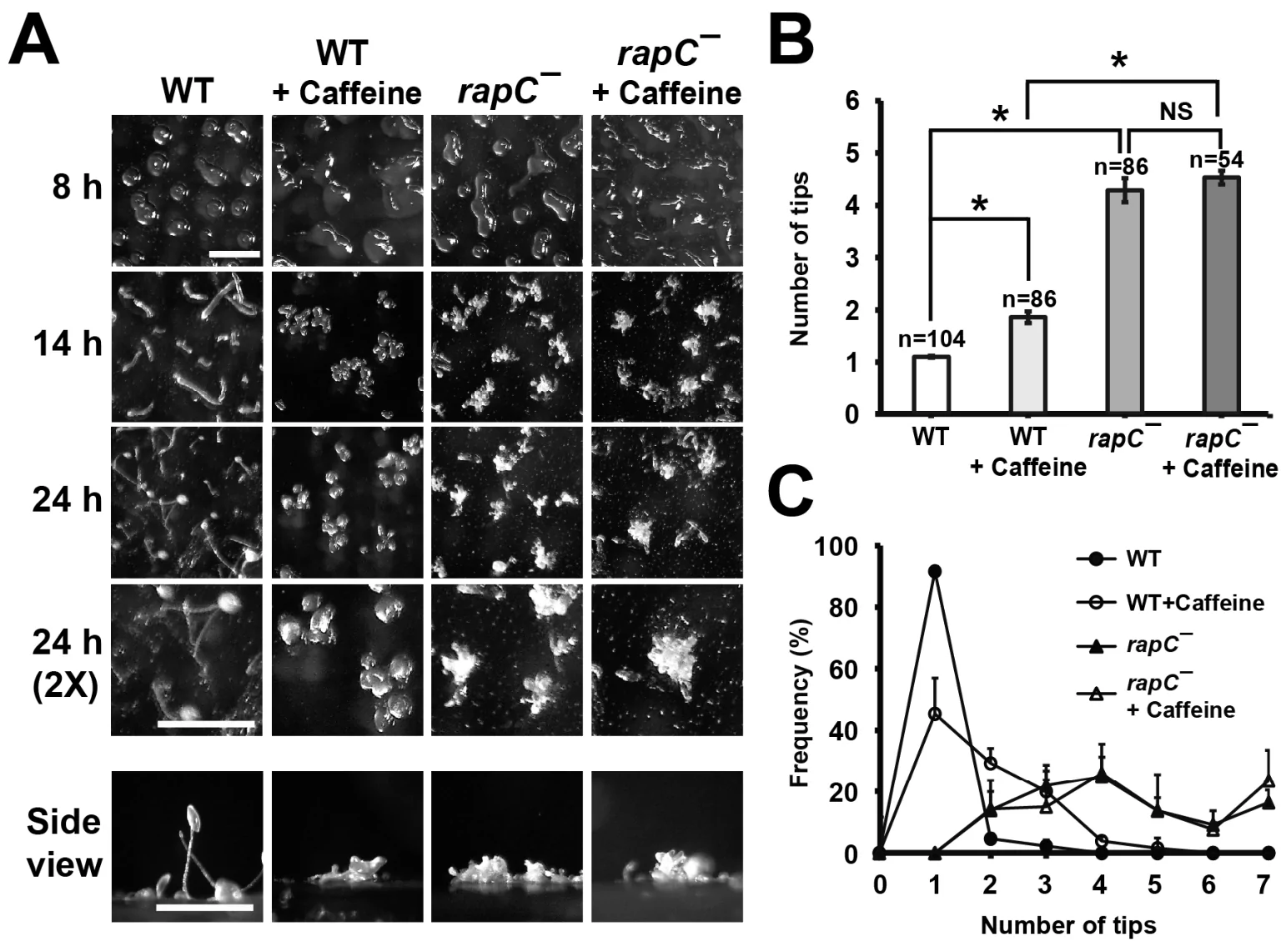
Caffeine treatment induces a multi-tip phenotype similar to *rapC* null cells. (**A**) Development of wild-type and *rapC* null cells in the presence of 2 mM caffeine. Representative images at 8, 14, and 24 h are shown. A magnified (2×) view of a portion of the 24 h image is shown directly below the corresponding panel. Side view of fruiting bodies at 24 h development. Scale bar = 0.5 mm. (**B**) Average number of tips per aggregate at 14 h. The graphs show the mean number of tips counted from multiple aggregates across three independent experiments. The number of aggregates analyzed (N) is indicated on each graph. Error bars indicate SD. One-way ANOVA with Tukey’s post hoc test was performed. NS, not significant; * *p* < 0.05, statistically significant. (**C**) Frequency distribution of tips per aggregate at 14 h.

Caffeine is widely used in *Dictyostelium* research to inhibit cAMP relay and has recently been shown to directly inhibit PI3K and TORC2 activity [25]. Caffeine has also been shown to induce multi-tip formation during *Dictyostelium* development [5]. To determine whether caffeine treatment phenocopies the *rapC* null developmental defect, we developed wild-type and *rapC* null cells in the presence of 2 mM caffeine. At 14 h, caffeine-treated wild-type cells formed multiple tips, with an average of approximately two tips per aggregate, representing a significant increase over untreated wild-type controls (Figure 2A,B). *rapC* null cells developed in caffeine displayed an additive phenotype, averaging 4–5 tips per aggregate (Figure 2A,B). Statistical comparison confirmed that the tip number in caffeine-treated *rapC* null cells was significantly greater than in caffeine-treated wild-type cells (Figure 2B), indicating that RapC loss and caffeine treatment exert partially additive rather than fully overlapping effects on tip dominance. The distribution of tip numbers in caffeine-treated wild-type cells closely resembled that of untreated *rapC* null cells, while caffeine treatment of *rapC* null cells produced a further shift toward higher tip numbers (Figure 2C). The partial additivity of the two perturbations indicates that caffeine treatment and loss of RapC do not act through entirely independent mechanisms. This observation motivates the use of more specific pharmacological tools to dissect the signaling pathway downstream of RapC.

### 3.3. TORC1 Inhibition by Rapamycin, but Not TORC2 or PI3K Inhibition, Phenocopies the rapC Null Multi-Tip Phenotype

Caffeine is a pleiotropic inhibitor with documented effects on PI3K, TORC2, and cAMP phosphodiesterase [25]. To identify the specific signaling branch whose disruption causes multi-tip formation, we employed three pharmacological agents that selectively target distinct nodes: LY294002 (PI3K inhibitor), Torin2 (TORC1/TORC2 dual inhibitor, primarily TORC2-active at the concentrations used for developmental assays), and rapamycin (selective allosteric TORC1 inhibitor). By comparing the developmental phenotypes produced by each agent, we aimed to determine whether the *rapC* null multi-tip defect reflects loss of PI3K, TORC2, or TORC1 activity.

**Figure 3:**
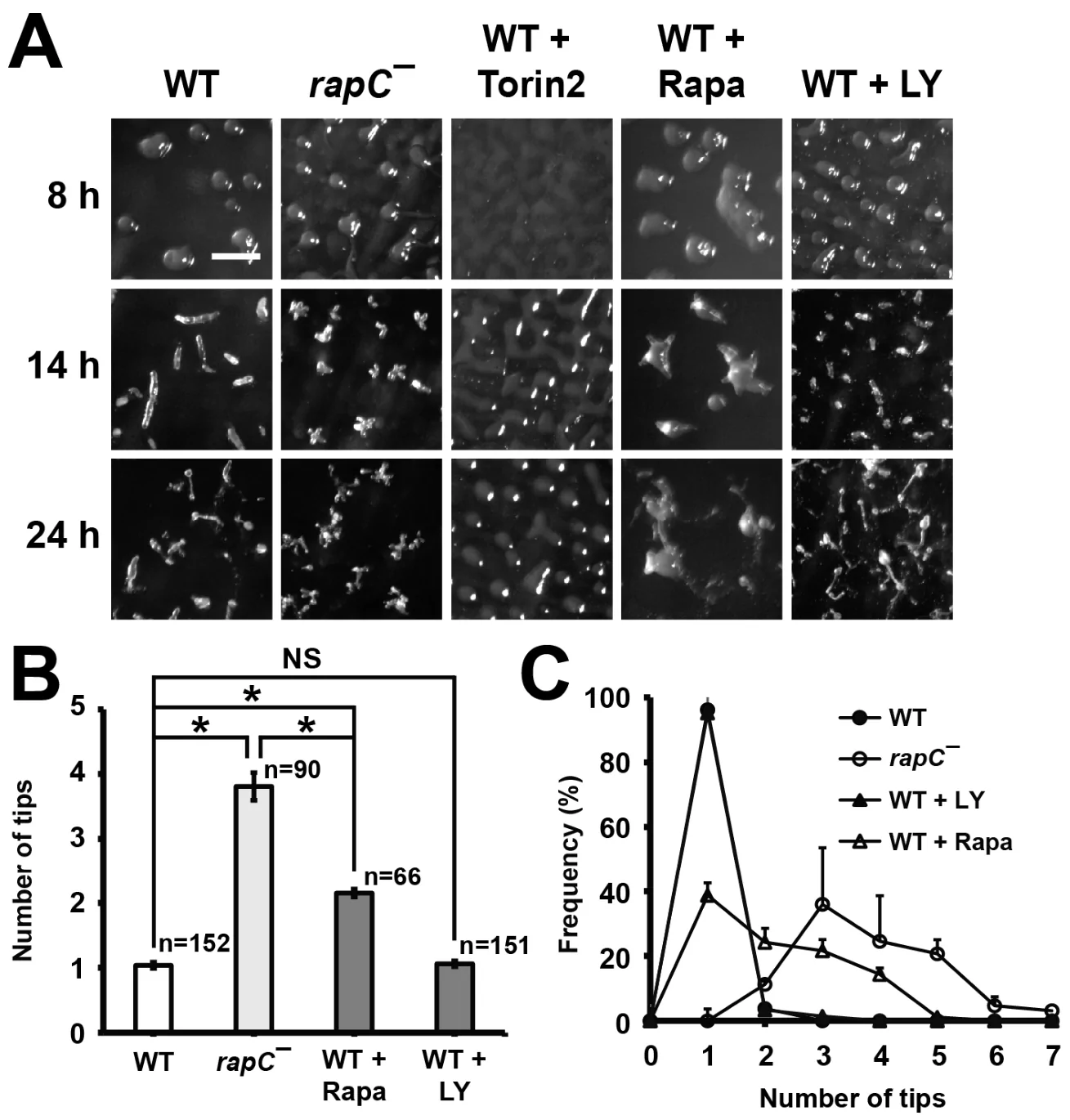
TORC1 inhibition by rapamycin phenocopies the *rapC* null multi-tip phenotype. (**A**) Development of wild-type cells in the presence of 30 µM LY294002 (PI3K inhibitor), 1 µM Torin2 (TORC1/2 inhibitor), or 50 nM rapamycin (TORC1 inhibitor). Wild-type and *rapC* null cells without inhibitor served as controls. Representative images at 8, 14, and 24 h are shown. Scale bar = 0.5 mm. (**B**) Average number of tips per aggregate at 14 h. The graphs show the mean number of tips counted from multiple aggregates across three independent experiments. The number of aggregates analyzed (N) is indicated on each graph. Error bars indicate SD. One-way ANOVA with Tukey’s post hoc test was performed. NS, not significant; * *p* < 0.05, statistically significant. (**C**) Frequency distribution of tips per aggregate at 14 h.

LY294002 (30 µM) treatment did not induce multi-tip formation. Wild-type cells in the presence of LY294002 developed with normal single-tip morphology comparable to untreated controls (Figure 3A–C), demonstrating that a defect requiring PI3K signaling is not the mechanism underlying the *rapC* null multi-tip phenotype. Torin2 (1 µM), which inhibits both TORC1 and TORC2, arrested development at the mound stage without producing tip emergence (Figure 3A), consistent with the established requirement of TORC2-mediated PKBR1 phosphorylation for mound-to-slug progression [11]. In striking contrast, rapamycin (50 nM), which selectively inhibits TORC1 while leaving TORC2-PKB signaling intact, induced a robust multi-tip phenotype in wild-type cells that qualitatively resembled the *rapC* null phenotype (Figure 3A–C). However, direct statistical comparison revealed that the average tip number in rapamycin-treated wild-type cells was significantly lower than in *rapC* null cells (Figure 3B), indicating that pharmacological TORC1 inhibition reproduces the qualitative multi-tip phenotype but does not fully recapitulate its magnitude. These results implicate TORC1 signaling in the restriction of tip number during development and suggest that RapC supports TORC1 activity to maintain tip dominance in the mound, while indicating that additional RapC-dependent mechanisms may contribute to the full penetrance of the *rapC* null phenotype.

### 3.4. rapC Null Cells Maintain Normal PI3K–TORC2–PKB Signaling

The finding that TORC2 inhibition arrests development but TORC1 inhibition produces multi-tip formation raises the question of whether *rapC* null cells have any perturbation in the TORC2-PKB chemotaxis signaling axis. If RapC loss compromised TORC2-dependent PKB activity, the developmental defect might reflect impaired cAMP relay rather than a TORC1-specific mechanism. To distinguish these possibilities, we directly quantified PI3K/PIP3 production and TORC2-dependent PKB/PKBR1 phosphorylation in *rapC* null cells.

**Figure 4:**
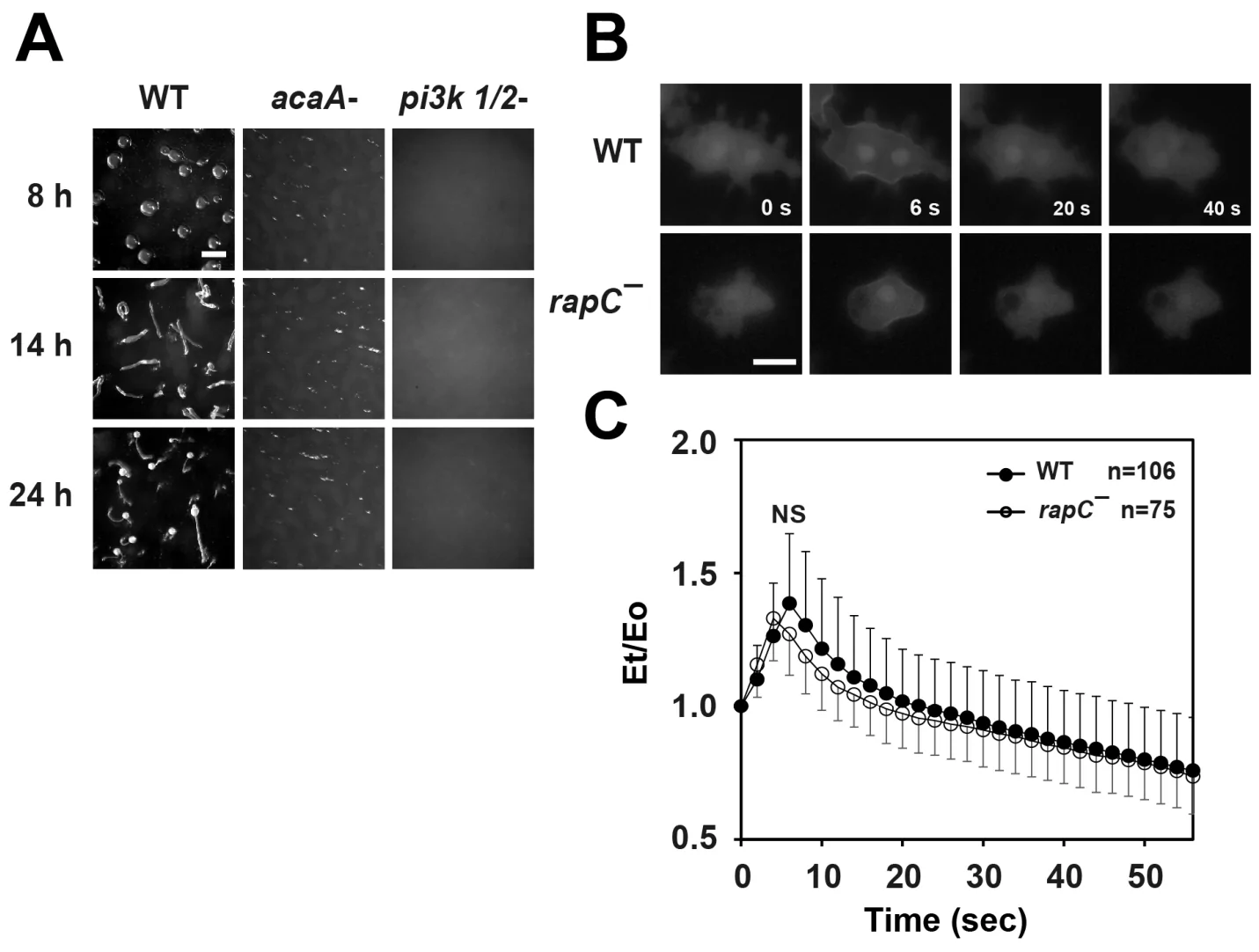
*rapC* null cells display normal PI3K signaling. (**A**) Development of *acaA* null and *pi3k1/2* null cells on non-nutrient agar (negative developmental controls). Representative images at 8, 14, and 24 h are shown. Scale bar = 0.5 mm. (**B**) Translocation of GFP-PH:CRAC to the cell cortex in response to uniform cAMP chemoattractant stimulation. Aggregation-competent wild-type and *rapC* null cells expressing GFP-PH:CRAC were stimulated with cAMP, and time-lapse images were recorded. Representative fluorescence images at 0, 6, 20, and 40 s are shown. Scale bar = 5 µm. (**C**) GFP-PH:CRAC translocation kinetics quantified as cortical fluorescence intensity (Et/E0) from time-lapse recordings following cAMP stimulation. The graphs represent the mean values ± SD of multiple cells from three independent experiments. The number of cells analyzed (N) is indicated. Two-tailed Student’s *t*-test at 6 s post-stimulation was performed. NS, not significant.

As controls, we confirmed that *acaA* null and *pi3k1/2* null cells fail to undergo normal multicellular development (Figure 4A), establishing the requirement of ACA-mediated cAMP relay and PI3K activity for developmental progression beyond aggregation. We then examined whether PI3K–TORC2–PKB signaling is perturbed in *rapC* null cells, which could potentially account for the developmental defect. Monitoring of GFP-PH:CRAC translocation in response to uniform cAMP stimulation revealed that aggregation-competent *rapC* null cells display cortical translocation kinetics indistinguishable from those of wild-type cells, achieving comparable peak Et/E0 values with identical temporal kinetics (Figure 4B,C). These data indicate that *rapC* null cells retain normal PIP3 production and PI3K activity in response to cAMP.

**Figure 5:**
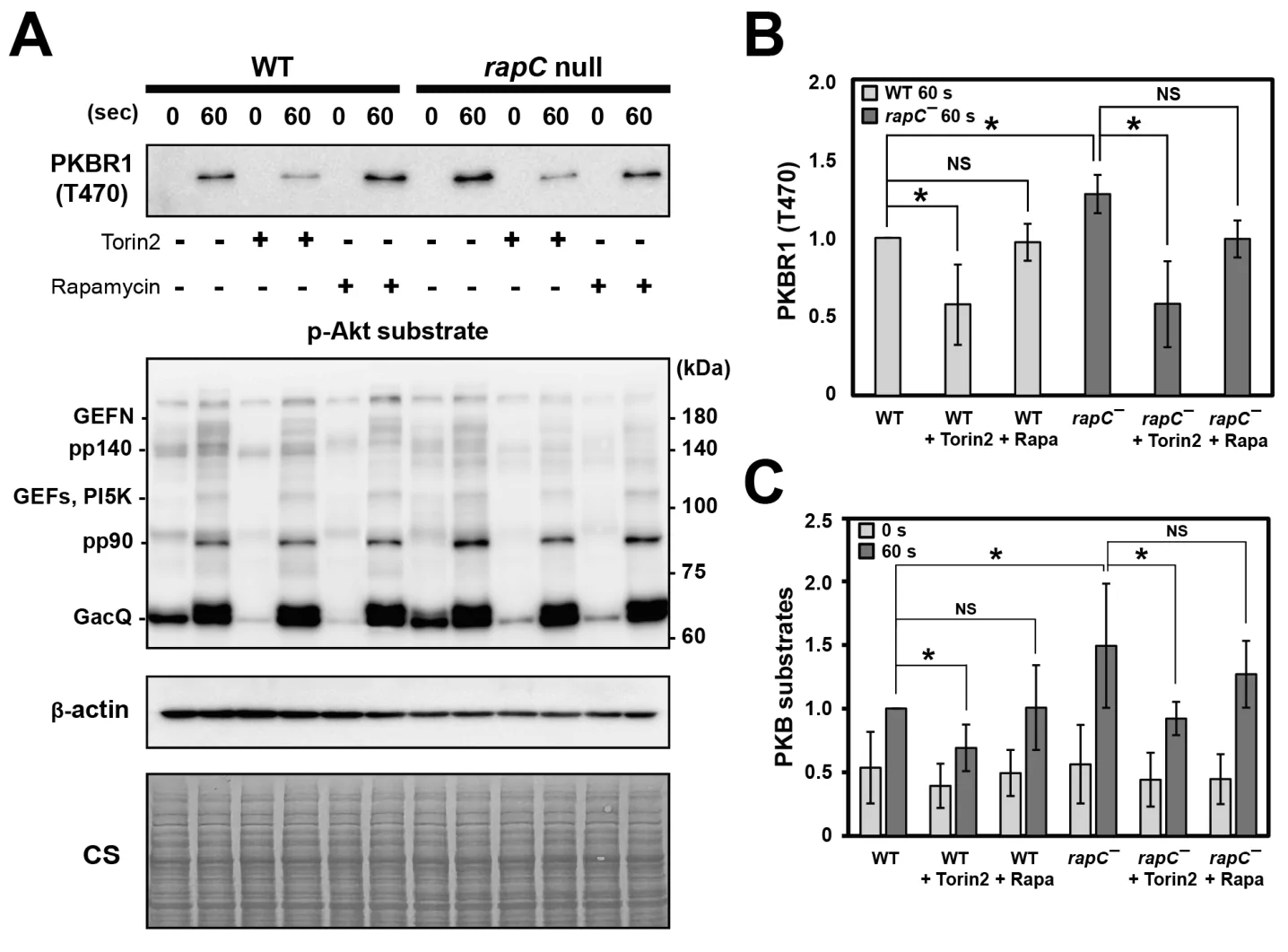
Normal or slightly elevated PKB/PKBR1 signaling in *rapC* null cells. (**A**) Immunoblot analysis of PKBR1 T470 phosphorylation and total PKB substrate phosphorylation following cAMP stimulation (0 or 60 s) in wild-type and *rapC* null cells with or without Torin2 or rapamycin. Phosphorylation of T470 in the hydrophobic motif of PKBR1 was detected using anti-phospho-p70 S6 kinase antibodies (upper panel) and phosphorylation of PKB substrates was detected using anti-phospho-Akt substrate antibodies (middle panel) that recognize R-X-R-X-X-S/T-X-X in a phosphorylation-dependent manner (R, arginine; S, serine; T, threonine; X, any amino acid). The proteins observed at pp180, pp110, and pp65/67 were identified as GEFN, GEFs and PI5K, and GacQ, respectively [11], and are marked on the left side of the panel. β-actin and Coomassie blue-stained blots serve as loading control (lower panel). (**B**) Quantification of PKBR1 T470 phosphorylation. The intensity of each band in the blots of PKBR1 phosphorylation was quantified from three independent experiments. (**C**) Quantification of PKB substrate phosphorylation. The combined intensities of the bands corresponding to GEFs and PI5K, pp90, and GacQ on the blots were quantified. The level of PKBR1 T470 phosphorylation or PKB substrate phosphorylation at 60 s after stimulation in wild-type cells was set to 1.0 and the others are relative amounts. Data represent mean ± SD from three independent experiments. One-way ANOVA with Tukey’s post hoc test was performed. NS, not significant; * *p* < 0.05, statistically significant.

We further assessed TORC2-mediated PKB signaling by immunoblotting for PKBR1 hydrophobic motif phosphorylation (T470) and total PKB substrate phosphorylation following cAMP stimulation [11]. Both wild-type and *rapC* null cells exhibited robust, kinetically similar cAMP-induced PKBR1 T470 phosphorylation, with *rapC* null cells showing similar or modestly elevated levels relative to wild-type (Figure 5B,C). Torin2 treatment reduced PKBR1 hydrophobic motif phosphorylation in both strains, confirming TORC2-dependent regulation, while rapamycin had comparatively little effect on hydrophobic motif phosphorylation, consistent with TORC1 selectivity (Figure 5B). These results collectively demonstrate that the PI3K–TORC2–PKBR1 axis is functionally normal in *rapC* null cells, and that the multi-tip phenotype of *rapC* null cells does not arise from defects in the PI3K-TORC2-PKB signaling axis.

### 3.5. rapC Null Cells Exhibit Elevated Autophagic Flux

Given the phenotypic similarity between *rapC* null cells and rapamycin-treated wild-type cells, and because TORC1 is the master negative regulator of autophagy [10,13], we hypothesized that autophagic flux is chronically elevated in *rapC* null cells. Using the RFP-GFP-Atg8 tandem reporter, we monitored autophagy in wild-type and *rapC* null cells under nutrient-rich (HL5) and starvation (DB, 4 h) conditions. Autophagosomes emit both red and green light and appear as yellow puncta, while the autolysosomes show red but not green puncta [19].

**Figure 6:**
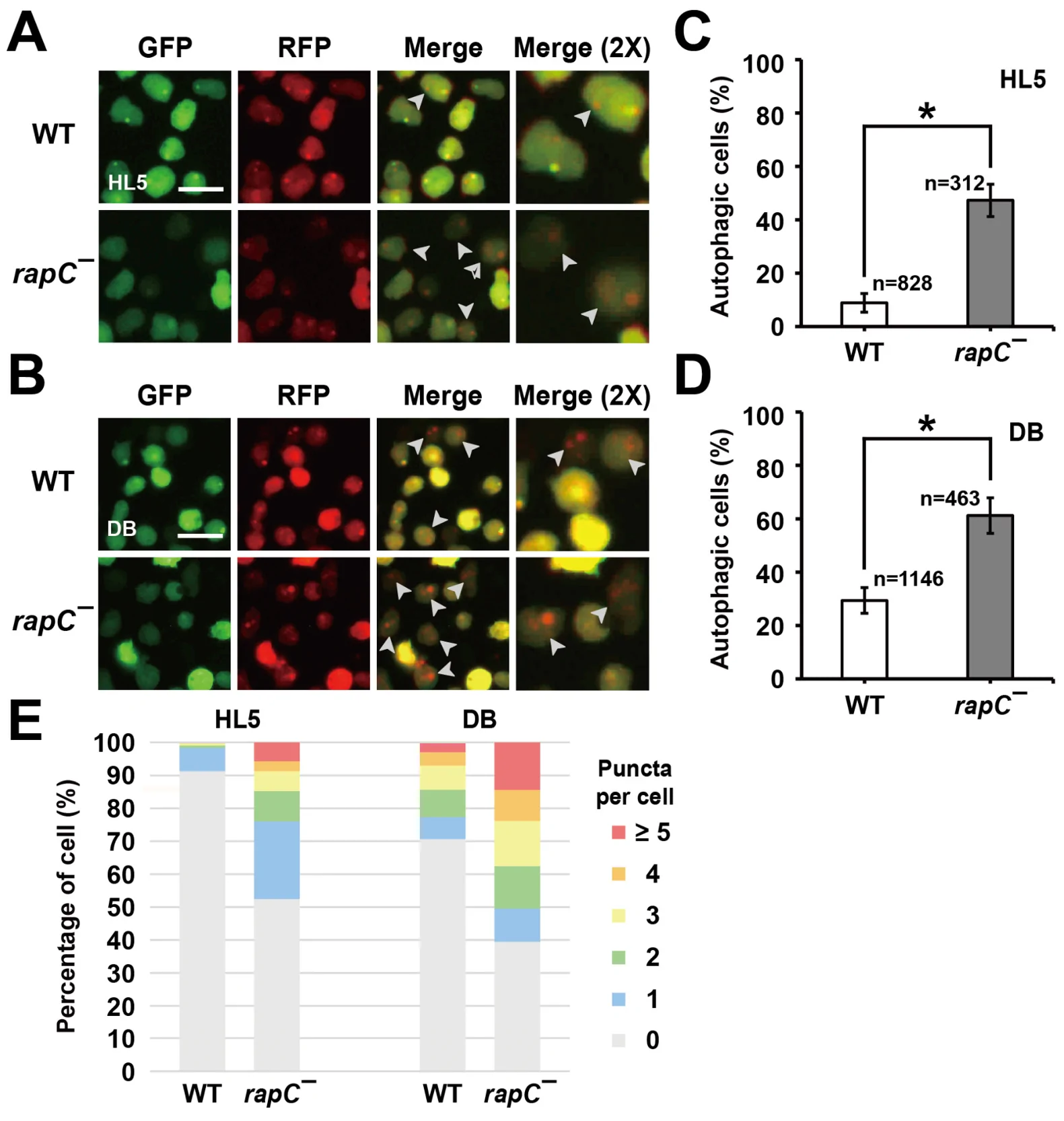
*rapC* null cells display elevated autophagic flux. Wild-type and *rapC* null cells stably expressing RFP-GFP-Atg8 were imaged during exponential growth (HL5 medium) or after 4 h of starvation (DB buffer). (**A**,**B**) Representative fluorescence images under HL5 (**A**) or DB (**B**) conditions. The yellow (due to both red and green light) and red-only (due to red but not green, arrowheads) puncta represent autophagosomes and autolysosomes, respectively. For clarity, a magnified (2×) image of a portion of the merged image is shown immediately to the right of each merged panel, labeled “Merge (2X)”. Scale bar = 10 µm. (**C**,**D**) Quantification of the autophagic cells under nutrient-rich (**C**) or starvation conditions (**D**). Autophagic cells were defined by the presence of red-only puncta. The percentage of autophagic cells was calculated by dividing the number of total cells containing green or red puncta by the number of cells containing red-only puncta. Data represent mean ± SD from at least three independent experiments. The number of cells analyzed (N) is indicated on each graph. Two-tailed Student’s *t*-test was performed. Statistically significant * *p* < 0.05. (**E**) Quantification of puncta per cell in wild-type cells and *rapC* null cells under HL5 and DB conditions.

Under nutrient-rich (HL5) conditions, where TORC1 is normally active, wild-type cells showed mostly overlapping GFP and RFP fluorescence (yellow puncta), whereas *rapC* null cells showed a significantly increased number of red-only puncta (Figure 6A). The percentage of autophagic cells was significantly higher in *rapC* null cells (approximately 50%) than in wild-type cells (approximately 10%) (Figure 6C). After 4 h of starvation in DB buffer, which normally triggers autophagy induction in wild-type cells via TORC1 suppression, both strains showed increased autophagic activity, but *rapC* null cells (approximately 60%) again exhibited significantly higher levels than wild-type cells (approximately 30%) (Figure 6B,D). The number of autophagic puncta per cell was also significantly greater in *rapC* null cells under HL5 or DB conditions (Figure 6E). Most wild-type cells contained few puncta (0 to 1 puncta per cell), whereas *rapC* null cells showed a higher proportion of cells containing multiple puncta (2 or more puncta per cell), and this difference was more pronounced under starvation conditions. These results demonstrate that RapC suppresses autophagic flux and that chronic upregulation of autophagy is a defining feature of *rapC* null cells, consistent with a deficiency in TORC1-mediated autophagy suppression.

### 3.6. rapC Null Cells Display Enhanced Intracellular Calcium Responses to External Stimulation

Ca^2+^ signaling has been linked to autophagy regulation [10,21], and Ca^2+^ transients are coupled to cAMP oscillations during *Dictyostelium* aggregation [1,22]. We therefore asked whether [Ca^2+^]i dynamics are aberrantly regulated in *rapC* null cells. Critically, comparing the calcium phenotype of *rapC* null cells with that of rapamycin-treated wild-type cells allows us to determine whether the calcium dysregulation is TORC1-dependent (rapamycin would reproduce it) or TORC1-independent (rapamycin would not reproduce it), thereby dissecting the two downstream arms of RapC function.

**Figure 7:**
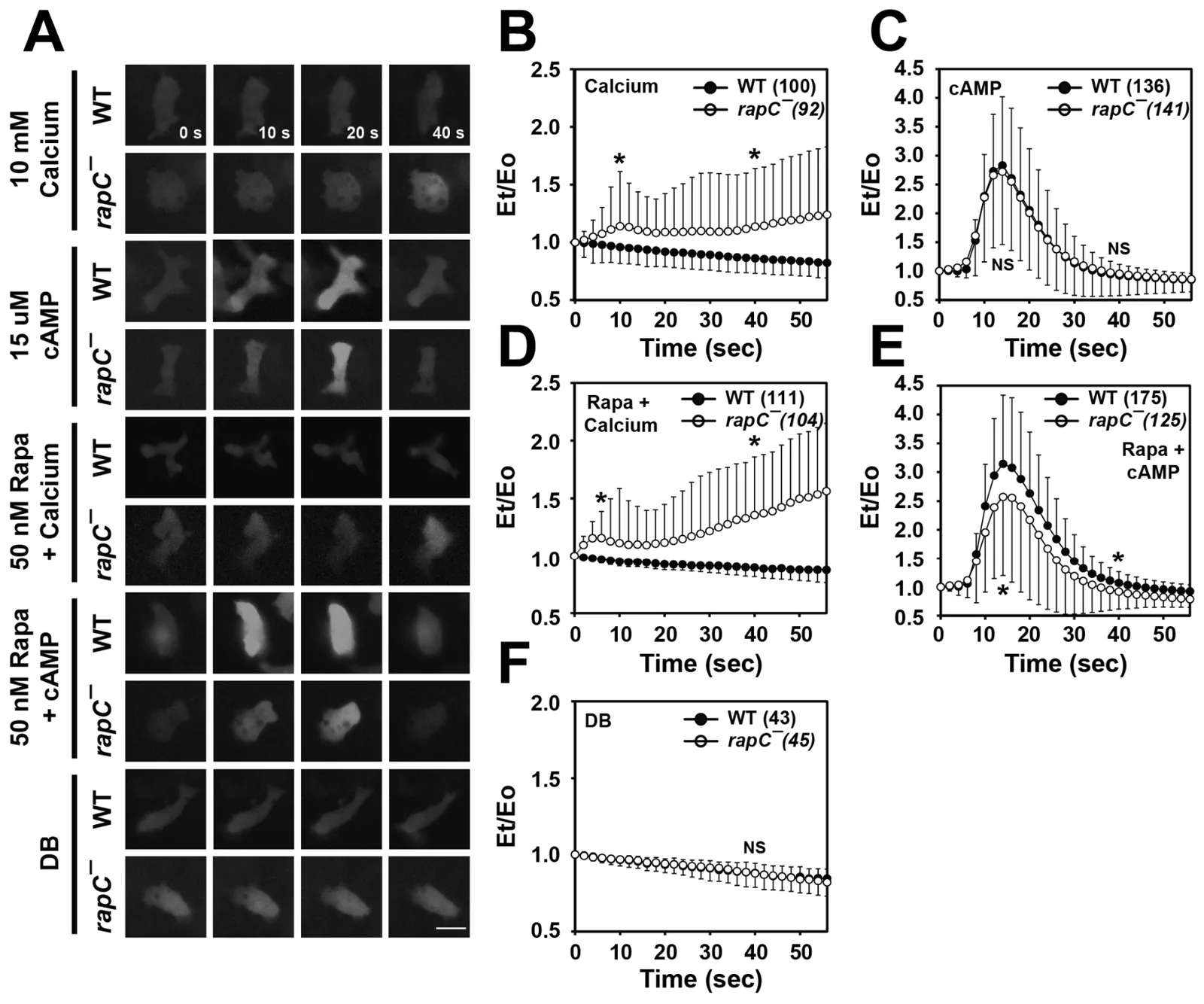
*rapC* null cells display enhanced intracellular calcium responses to external stimulation. Aggregation-competent wild-type and *rapC* null cells expressing GCaMP6 in the absence or presence of rapamycin were uniformly stimulated with 10 mM CaCl_2_ or 15 µM cAMP. (**A**) Representative GCaMP6 fluorescence images of wild-type and *rapC* null cells at the indicated time points (0, 10, 20, 40 s) following calcium or cAMP stimulation. Scale bar = 5 µm. (**B**,**C**) [Ca^2+^]i dynamics expressed as Et/E0 following external calcium (**B**) or cAMP (**C**) stimulation. (**D**,**E**) [Ca^2+^]i dynamics in cells pre-treated with 50 nM rapamycin for 30 min, followed by calcium (**D**) or cAMP (**E**) stimulation. (**F**) [Ca^2+^]i dynamics in wild-type and *rapC* null cells under starvation (DB) conditions without any stimulation. The graphs represent the mean values ± SD of multiple cells from three independent experiments. The number of cells analyzed (N) is indicated in parentheses next to the legend. Two-tailed Student’s *t*-test was performed. NS, not significant; * *p* < 0.05, statistically significant. Statistically significant differences were detected between wild-type and *rapC* null cells at 10 and 40 s post-calcium stimulation (**B**) and at 8 and 40 s in the presence of rapamycin (**D**), and between cAMP responses with and without rapamycin at 14 and 40 s (**E**); no significant differences were detected in panels (**C**,**F**). NS, not significant.

Using aggregation-competent cells expressing the genetically encoded Ca^2+^ indicator GCaMP6, we monitored [Ca^2+^]i responses to uniform stimulation with external CaCl_2_ (10 mM) or cAMP (15 µM). Aggregation-competent wild-type cells showed no detectable [Ca^2+^]i increase upon external calcium stimulation (Figure 7A,B), indicating that calcium entry and/or intracellular calcium release is actively suppressed at this developmental stage in wild-type cells [24]. In contrast, *rapC* null cells responded to the same calcium stimulus with a rapid [Ca^2+^]i elevation, reaching peak Et/E0 values approximately 2.5-fold above baseline (Figure 7A,B). There was no difference in [Ca^2+^]i responses upon cAMP stimulation between wild-type and *rapC* null cells (Figure 7C). These data reveal that RapC is required for the developmental suppression of Ca^2+^ responsiveness upon external calcium stimulation in aggregation-competent cells.

To determine whether the calcium response of *rapC* null cells is a downstream consequence of reduced TORC1 activity, we pre-treated aggregation-competent wild-type cells with 50 nM rapamycin for 30 min prior to calcium or cAMP stimulation. Rapamycin-treated wild-type cells failed to exhibit a detectable [Ca^2+^]i increase in response to external calcium stimulation (Figure 7D), indicating that TORC1 inhibition alone is insufficient to reproduce the calcium phenotype of *rapC* null cells. There was no significant difference in calcium responses of *rapC* null cells to external calcium or cAMP stimulation in the presence and absence of rapamycin (Figure 7D,E). These findings demonstrate that RapC controls calcium homeostasis through a TORC1-independent mechanism, and that RapC coordinates *Dictyostelium* development through at least two mechanistically distinct signaling pathways: a TORC1-dependent autophagy regulatory branch and a separate, TORC1-independent calcium homeostatic branch. Quantitative comparison confirmed statistically significant differences between wild-type and *rapC* null calcium responses at 10 s and 40 s following external calcium stimulation (Figure 7B) and at 8 s and 40 s in the presence of rapamycin (Figure 7D), and between cAMP-stimulated responses with and without rapamycin at 14 s and 40 s (Figure 7E), whereas no significant differences were detected upon cAMP stimulation alone (Figure 7C) or under unstimulated starvation control conditions (Figure 7F), confirming the specificity of the calcium phenotype to external calcium stimulation and its independence from TORC1 activity.

## 4. Discussion

In this study, we characterized the developmental function of RapC, a Rap-family small GTPase, in *Dictyostelium discoideum*. Our central findings are as follows: (i) *rapC* null cells form multiple apical tips during mound morphogenesis, fully rescued by GFP-RapC re-expression; (ii) the multi-tip phenotype is phenocopied by caffeine and the TORC1-selective inhibitor rapamycin, but not by PI3K or TORC2-inclusive inhibition; (iii) PI3K–TORC2–PKB signaling is intact in *rapC* null cells; (iv) *rapC* null cells exhibit elevated autophagic flux under both nutrient-rich and starvation conditions, consistent with reduced TORC1 activity; and (v) aggregation-competent *rapC* null cells display a [Ca^2+^]i response to external calcium that is absent in wild-type cells and independent of TORC1. Collectively, these results establish RapC as a novel regulator of multicellular morphogenesis acting through two distinct downstream branches, TORC1–autophagy and calcium homeostasis.

The multi-tip phenotype of *rapC* null cells indicates a defect in tip dominance, the process by which the initial organizing tip generates inhibitory signals that prevent the emergence of secondary tips. This mechanism relies on spatially coherent oscillatory cAMP relay and on the tip-specific restriction of ACA–PKA signaling [1,2]. Compared with previously characterized multi-tip mutants, the *rapC* null phenotype has a distinct mechanistic basis. *5nt* null cells lose tip dominance through defective extracellular cAMP gradient maintenance [7]; TagA truncation mutants overproduce prestalk cells, creating supernumerary organizing centers from cell fate imbalance [4]; and SunB loss disrupts cell division and developmental gene expression [8]. Our data indicate that *rapC* null cells lose tip dominance through TORC1 attenuation, independently of PI3K–TORC2–PKB signaling perturbation, placing RapC as a novel nutrient-sensing input into the tip dominance mechanism.

The selective induction of multi-tip formation by rapamycin, but not by Torin2 which inhibits both TORC1 and TORC2, reveals that TORC1 and TORC2 play functionally distinct roles in mound morphogenesis. TORC2 is required for the mound-to-slug transition, consistent with PKBR1-mediated chemotaxis signaling [11], whereas TORC1 specifically restricts tip number within the mound. The mechanistic convergence of rapamycin treatment and loss of RapC on both multi-tip formation and elevated autophagy provides strong evidence that RapC positively regulates TORC1 activity during the aggregation-competent and mound stages of development. This conclusion is corroborated by the selective sensitivity of PKBR1 HM phosphorylation to Torin2, but not to rapamycin. This result confirms that TORC2 signaling is intact in *rapC* null cells and is not the causal mechanism of the developmental phenotype.

The elevated autophagic flux in *rapC* null cells is consistent with chronic TORC1 attenuation, as TORC1 normally suppresses autophagy by phosphorylating the Atg1/ULK1 kinase complex [13,18]. Constitutive autophagy activation may impair tip dominance through excessive degradation of tip-inhibitory signaling components, perturbation of energy homeostasis, or disruption of intercellular secretome signaling. Notably, caffeine phenocopies both the multi-tip and elevated autophagy phenotypes. Caffeine has been shown to inhibit PI3K and TORC2 in *Dictyostelium* [25], suggesting that caffeine and rapamycin may converge on TORC1 suppression through distinct upstream mechanisms to deregulate tip dominance.

The enhanced [Ca^2+^]i response of *rapC* null cells to external calcium is striking, as aggregation-competent wild-type cells are completely refractory to the same stimulus [24]. This stage-specific gating of calcium responsiveness suggests that calcium entry and/or intracellular release is actively suppressed during aggregation, and that RapC is required for this gating. Crucially, rapamycin treatment fails to reproduce the enhanced calcium response, demonstrating that this function of RapC is independent of TORC1. In mammalian systems, elevated cytosolic calcium can suppress mTORC1 via CaMKKβ–AMPK [21], raising the possibility that dysregulated calcium in *rapC* null cells may secondarily amplify TORC1 suppression, thereby reinforcing the autophagy and multi-tip phenotype.

We note two limitations of the present study that require further investigation. First, although rapamycin treatment of wild-type cells reproduces the qualitative multi-tip phenotype of *rapC* null cells, quantitative comparison revealed that the average tip number differs significantly between the two conditions (Figure 3B), indicating that pharmacological TORC1 inhibition alone does not fully phenocopy loss of RapC. Establishing a direct genetic link between RapC and TORC1 pathway components, for example by testing whether overexpression of TORC1 pathway genes rescues the *rapC* null developmental defect, or whether constitutively active RapC rescues rapamycin-treated wild-type cells, was beyond the scope of the present study and remains an important direction for future work to more rigorously define the RapC–TORC1 regulatory relationship.

Second, while our data establish that RapC loss alters intracellular calcium ([Ca^2+^]i) responses in aggregation-competent cells, they do not establish a direct causal link between this altered calcium response and the multi-tip developmental phenotype. The calcium and autophagy/TORC1 phenotypes are presented here as two mechanistically distinct, RapC-dependent consequences of RapC loss rather than as a single linear causal pathway to tip multiplication. Testing this causal relationship directly, for instance by supplementing extracellular calcium to determine whether it reverses the *rapC* null multi-tip phenotype, or by depleting intracellular calcium stores in wild-type cells to test whether this is sufficient to induce multi-tip formation, will be an important goal of future studies. In addition, whether re-expression of GFP-RapC also suppresses caffeine-induced multi-tip formation was not directly tested here and remains an informative test of pathway convergence for future work.

## Data Availability

The original contributions presented in this study are included in the article. Further inquiries can be directed to the corresponding authors.
